# Nanotechnology for the detection and kill of circulating tumor cells

**DOI:** 10.1186/1556-276X-9-500

**Published:** 2014-09-15

**Authors:** Yang Gao, Zhou Yuan

**Affiliations:** 1 Department of Surgery, Shanghai Jiao Tong University Affiliated Sixth People's Hospital, Shanghai 200233, China

**Keywords:** Nanotechnology, Circulating tumor cell, Metastasis, Detection, Prognosis

## Abstract

Circulating tumor cells (CTCs) represent a surrogate biomarker of hematogenous metastases and thus could be considered as a ‘liquid biopsy’ which reveals metastasis in action. But it is absolutely a challenge to detect CTCs due to their extreme rarity. At present, the most common principle is to take advantage of the epithelial surface markers of CTCs which attach to a specific antibody. Antibody-magnetic nanobeads combine with the epithelial surface markers, and then the compound is processed by washing, separation, and detection. However, a proportion of CTC antigen expressions are down-regulated or lost in the process of epithelial-mesenchymal transition (EMT), and thus, this part of CTCs cannot be detected by classical detection methods such as CellSearch. To resolve this problem, some multiple-marker CTC detections have been developed rapidly. Additionally, nanotechnology is a promising approach to kill CTCs with high efficiency. Implantable nanotubes coated with apoptosis-promoting molecules improve the disease-free survival and overall survival. The review introduces some novel CTC detection techniques and therapeutic methods by virtue of nanotechnology to provide a better knowledge of the progress about CTC study.

## Review

### Introduction

Circulating tumor cells (CTCs) are shed from primary tumors, enter the circulatory system, and migrate to distant organs to form metastases that ultimately lead to the death of most cancer patients [1]. It is of great importance to carry out further study on CTCs because tumor is the major cause of human beings' death and approximately 90% of death is caused by the secondary tumor [2-4].

Thomas Ashworth firstly proposed the hypothesis of CTCs in 1869 [5], but studies on CTCs have made little progress in the last century mainly due to the limited condition to detect the CTCs. It is a challenging job to detect the CTCs in the peripheral blood since the CTCs are extremely scarce (just a few CTCs mixed with the approximately 10 million leukocytes and 5 billion erythrocytes in 1 ml of blood) [6]. The current studies indicate that there is a close relationship between CTC enumeration and the severity of disease and clinical prognosis, thus initiating further studies. The CTC number has been established as a helpful prognostic biomarker of malignant epithelial tumor including breast cancer [7,8], prostate cancer [9,10], and colon cancer [11]. With the relative technology developed rapidly, the achievements about CTCs are fruitful in the last decade.

The metastatic process is comprised of the following steps: neoangiogenesis, intravasation, circulation in the peripheral blood, extravasation, and ultimately resulting in colonization and growth at distant sites [12,13]. Obviously, CTCs act as the ‘vector’ of metastasis, and the successful capture and analysis of the CTCs throw light on the nature of the primary tumor and characterization of the secondary tumor. However, tumor cells in circulation are heterogeneous. On the one hand, they can exist in the circulatory system in various forms, such as simple cells, clumps of tumor cells, association with blood cells, and non-viable cells, so the CTCs are only a part of the tumor cells in circulation and other forms of tumor cells can also form metastasis [14]. On the other hand, during invasion and metastasis in epithelial tumors, epithelial-mesenchymal transition (EMT), a phenotypic change in epithelial cells may occur. Tumor cells lose the normal cell-to-cell interactions and adopt a more mesenchymal phenotype in the process of EMT, which facilitates their invasion through the vascular endothelium and migration into the circulation. Then CTCs extravasate out of the circulation and invade into tissues to ultimately form a metastasis, and the reverse progress is called mesenchymal-epithelial transition (MET). The epithelial surface markers alter during the process of EMT and MET, so antibody-based methods will lose some malignant tumor cells [14,15]. In addition, there is no single epithelial marker which is expressed in all tumor cells. Although the epithelial cellular adhesion molecule (EpCAM) is the most used tumor surface biomarker in the detection of CTCs, it is not necessarily expressed in all types of epithelial tumor. Furthermore, EpCAM may be lost in the process of EMT, so some CTCs can be missed by CellSearch. Thus, we should take the above properties into account when applying new technologies to detect and manipulate the CTCs. Therefore, simultaneous staining with multiple epithelial markers or negative staining with leukocyte markers is the future trend to detect CTCs.

Nanotechnology, the manipulation of matter on an atomic, molecular, or supramolecular scale, is expected to show great capability for the early detection, accurate diagnosis, and personalized treatment of malignant tumors. Nanoscale particles are typically about several hundreds of nanometers or smaller and are approximately equal to the size of large biological molecules such as enzymes, receptors, and antibodies. These nanoscale devices interact with biomolecules both on the surface of and inside cells with unprecedented efficiency, which may improve disease diagnosis and treatment drastically [16,17]. Many new technologies are derived from the application of nanotechnology, for example, CellSearch System [18], AdnaSystem [19], versatile immunomagnetic nanocarrier platform [20], one-step detection method [21], aptamer application [22], and magnetophoresis technology [23] (Table 1). Nanoscale devices have several advantages over other devices: high sensitivity, microsize, portability, and availability of point-to-care medicine, which will play a crucial role in future translational medicine.

**Table 1:** Characteristics of some CTC detection methods

**Method**	**Cell viability**	**Detection level**	**Advantage**	**Disadvantage**	**Reference**
CellSearch System	No	Median number of isolated CTCs, 5 CTCs per 7.5 ml of blood	Adequate clinical evidence, automated enumeration, commercial availability	Further analysis limited, false-positive and false-negative, applications need to be expanded	[18,25,26]
CellSearch Profile	No	Median number of isolated CTCs, about 140 CTCs per 7.5 ml of blood	Fewer processing steps, better sensitivity, reproducibility	Manual enumeration, false-positive and false-negative, limited reports	[32]
AdnaSystem	No	Sensitivity, 2 CTCs per 5 ml of blood	Detection of occult or very low number of CTCs, assessment of genomic markers	False-positive and false-negative, the sensitivity not improved compared to CellSearch System	[19,37]
Immunomagnetic nanocarrier platform	Yes	Capture rate, 78% to 93%	High capture rate, fewer processing steps, altered biomarkers	Lacks clinical study, limited reports	[20]
Hybrid nanoparticle	Yes	Capture rate, 87.5%	High capture rate, fewer processing steps	Lacks clinical study, limited reports	[41]
One-step method	No	Capture rate, about 3/1,000	Convenient process, low cost	Low capture rate, lacks clinical data, false-negative	[21]
μ-Nuclear magnetic resonance	No	Capture rate, 99.2%	High sensitivity, short measurement time	Lacks clinical study	[42,43]
Aptamer-conjugated gold nanoparticles	No	The limit of detection is 90 cells	Bare eyes sense the color change, the detection is rapid	Unable enumeration, CTC detection is still few	[22,47]
CTC microseparator	Yes	Isolates about 90% of CTCs	The step is simple and high throughput, the further can be carried out	Lacks clinical study	[23,49,50]

The current methods of detection of CTCs mainly constitute three stages, and a corresponding technology is applied in every stage [24]. Due to the rarity of the CTCs, the blood sample should firstly be pretreated to eliminate background cells, such as erythrocytes and leukocytes, or the tumor cells should be roughly separated according to the physical properties or the biological properties. Then different techniques are applied to identify the ‘real CTC’ by different labeling methods in the cellular and genetic levels. Lastly, powerful analytical instruments are required to analyze the superficial or interior biomarkers of the cell and the expression level of relative oncogenes and anti-oncogenes. Classical detection methods usually carry out the three stages in sequence, and nanoparticles prove to be a powerful tool in the process of CTC detection. Herein, nanotechnology will show its special property and function in the following nanoscale devices during the process of CTC detection.

### The application of nanotechnology in CTC detection

#### CellSearch System

CellSearch System, the current gold standard of CTC detection, is the only detective method approved by the US Food and Drug Administration (FDA) [18,25,26]. The CTC enumeration determined by CellSearch System predicts the disease-free survival (DFS) and overall survival (OS) in patients with breast cancer, colorectal cancer, and prostate cancer in multicenter studies, and it proves to be an independent predictive factor [7,11,27,28].

Although CellSearch System is the gold standard to detect CTCs, many limitations still exist yet. In 131 different histological tumor types and subtypes, 33.6% of CTCs have low or positive expression of EpCAM. Only 81% of adenocarcinomas of the colon, 78% of pancreas cancer, and 71% of hormone-refractory adenocarcinomas of the prostate have positive EpCAM expression [29]. Some tumor lines lose the expression of cytokeratins (CK8, CK18, and CK19) during metastasis [30]. Besides, during the treatment of cancer, if normal epithelial cells fall into the peripheral blood, they will mix up with CTCs and it will be difficult to identify them. In addition, the fact that many tumor cells with high-grade malignancy lose surface antigens during epithelial-mesenchymal transition (EMT) indicates that the detection of the antigen of epithelial cells may miss the most malignant tumor cells [6,15,31]. Furthermore, the separated CTCs undergoing centrifugation and permeabilization cannot be cultured and studied further during the process.

The amount of CTCs is also a limiting factor. The CTCs are so rare that 7.5 ml of blood sample of the total 5 l in an adult may not completely reflect the entire tumor cell population. More blood sample means higher positive detection. Of course, it is not a proper way to improve the sensitivity. Notably, the FDA-cleared CellSearch System may underestimate the number of CTCs; an investigative CellSearch Profile approach could nearly detect a 30-fold higher number of CTCs by using the same paired blood samples [32]. As more accurate and sensitive methods are developed, the CTCs can be tested with less blood sample and shorter turnaround time (a 30-min window is preferred) [33]. The current CellSearch System still cannot meet the need for a point-of-care test since the CTC assignment process is not operated as simple and automatic as the pregnancy test and diabetes test. As a new diagnostic method, the clinical significance of screening CTCs needs to be validated and more evidence should be accumulated to form the acknowledged guidelines and diagnosis criterion [24,34].

Therefore, application to routine clinical practice is still controversial. Many constitutions and guidelines are conservative to the application of the CTC enumeration, so more evidences should be collected to validate its utility in tumor diagnosis and treatment [35]. To overcome the limitations of CellSearch System, many new devices have been developed, although most of those are still in the experimental stage.

#### AdnaSystem

Due to the rarity of the CTCs in the peripheral blood, the detection technique must show high sensitivity, and reverse transcription polymerase chain reaction (RT-PCR) provides that sensitivity and feasibility [36]. AdnaTest BreastCancerSelect/Detect (AdnaGen AG, Langenhagen, Germany) is a new device to detect CTCs. The AdnaTest can detect as few as one tumor cell in 1 ml of mouse blood in a blood spiking experiment [37]. Human breast cancer cells are pre-enriched firstly by immunological nanobeads functionalized with three different antibodies, then the separated cells are lysed, and PCR is performed for HER2, MUC1, and GA733-2. Similarly, PCR for GA733-2, CEA, and epidermal growth factor receptor (EGFR) is performed to diagnose colon cancer and PCR for PSMA, PSA, and EGFR to diagnose prostate cancer [19,38]. Although the AdnaTest has equivalent sensitivity to that of CellSearch System in detecting two or more CTCs, it has several advantages over the latter [38]. Firstly, after recognition of tumor-associated markers, the isolated mRNA from CTCs can further be used for high-throughput gene expression profiling. Secondly, isolation and detection of stem cell and EMT markers can be realized. Thirdly, the CTC selection process and detection process could be used separately and may decrease the cost of detection of CTCs. However, we should put the shortcomings in mind: on the one hand, the AdnaTest is liable to present false-positive due to marker expression on non-tumor cells; on the other hand, enumeration of CTCs is not likely to undertake.

#### Versatile immunomagnetic nanocarrier platform

The biomarkers of CTCs have changed during EMT, for example, EpCAM might be down-regulated and expression of vimentin is present, so EpCAM-based methods miss some CTCs [39]. Supposing more useful markers are applied to detect the CTCs, the capture efficiency would be improved significantly. Deng et al. report that the assay method combining CK and EpCAM detects 15% to 111% more CTCs than the CellSearch™ method in patients with higher CTC counts (>20 CTCs per 7.5 ml of blood) [40].

**Figure 1:**
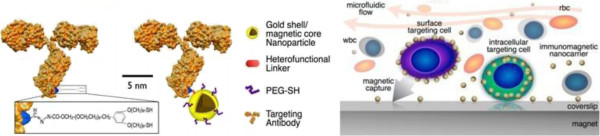
**Schematic of an antibody molecule combined with immunomagnetic particle and versatile immunomagnetic nanocarrier platform.** Left: schematic of an antibody molecule combined with an immunomagnetic particle. The nanoparticle is composed of a gold shell and an iron oxide core. The surface is functionalized with a heterofunctional linker and polyethylene glycol terminated with dithiol group (PEG-SH) which are used to link the antibody and the nanoparticle. Right: a versatile immunomagnetic nanocarrier platform in microfluidics for capturing CTCs. (Adapted from [20]).

The immunomagnetic nanocarrier platform is a nanoparticle with a gold shell and iron oxide core (Figure 1) [20]. The gold shell is coated with PEG-SH and a heterofunctional linker conjugated with a specific antibody directly. After adding magnetic nanobeads labeled with anti-EpCAM, anti-HER2, anti-EGFR, and anti-CK antibodies into different cell lines, the cell surface markers are identified by dark-field microscopy. The CTC capture efficiency is improved by combining microliquid technology to make full use of the intracellular and extracellular biomarkers. In a tumor cell spiking experiment, peripheral blood from healthy volunteers and a certain amount of CTCs are mixed up, and then the sample is passed through a microfluidic chamber at a continuous rate of 2.5 ml/h in the magnetic field and then identified using fluorescent staining. Notably, the CTC capture efficiency is 70% to 80% by using a nanoparticle coated with anti-CK antibody similar to the FDA-approved CellSearch System. But the CTC capture efficiency can be enhanced by utilizing two different nanoparticles. In addition, the combined tests improve the capture efficiency of EpCAM-low expressed cells. In this way, the EpCAM-low expressed cells are detected efficiently. The immunomagnetic nanocarrier platform captures CTCs efficiently, enumerates CTCs accurately, and analyzes the surface molecules in detail. The process eliminates multiple preprocessing steps such as plasma replacement, centrifugation, and sample transfer between tubes which are commonly used in other assays, for example, the CellSearch method. Thus, the simple and efficient platform is a promising CTC detection method.

Recently, Lee et al. demonstrate capture, *in situ* protein expression analysis, and cellular phenotype identification of CTCs simultaneously by using hybrid nanoparticles (HNPs) [41]. Each HNP constitutes three parts: antibodies that bind specifically to a known biomarker for CTCs, a quantum dot that emits fluorescence signals, and biotinylated DNA which is bound with streptavidin-coated quantum dots. They test three different breast cancer subtypes, and the average capture efficiency of CTCs is 87.5% with an identification accuracy of 92.4%. Subsequently, captured cells are released at efficiencies of 86.1% by cleaving the DNA portion with a restriction enzyme. Further study indicates that the released cells are viable and proliferative *in vitro*. The method has several advantages: it could efficiently capture heterogeneous CTCs, including those with low EpCAM expression; the progress without organic dye could not kill CTCs; and the ‘interested’ cells are selectively released and then cultured *in vitro*, enabling additional studies possible, such as drug screening and gene analysis. Although the above two methods seem to be perfect for capturing and analyzing CTCs, there is still a long way to apply the new technology to routine clinical practice.

#### Rapid detection method

During a series of processes, such as erythrocyte lysis, cell centrifugation, and washing, many CTCs get lost and much detection time and money are spent, so it is a trend to study rapid detection. Kim et al. [21] report a rapid one-step method to detect ovarian cancer CTCs. They develop a fluorescent nanoprobe with enhanced fluorescent intensity (magnetic NP [MNP]-SiO_2_ [rhodamine B isothiocyanate (RITC)]) and then combine it with the MUC1 monoclonal antibody. The compound is added into the sample directly, and after washing and fixation, approximately 10^7^ cells are analyzed in the FCM. The number of cells with a positive signal indicative of OVCAR-3 cells is counted as 23, 32, 58, and 387 for the unspiked blood samples and the indirect blood models with 100, 1,000, and 10,000 OVCAR-3 cells, respectively. This method needs no enrichment and fewer washing, and it also detects CTCs rapidly and feasibly. But the method should be validated via clinical trials. Obviously, the capture rate is so low that the application is limited too much; if more specific monoclonal antibodies are attached to the magnetic nanoparticles, the result will be more satisfying.

**Figure 2:**
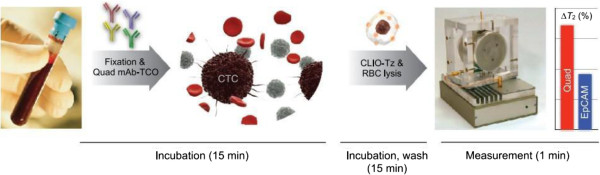
**Schematic of the quad-μNMR system.** After being incubated with four different mono-antibodies, the sample is spun down in the μNMR device and the sensitivity is improved significantly. (Adapted from [43]).

Micro-nuclear magnetic resonance (μNMR) is a novel method to detect circulating tumor cells; it consists of solenoidal microcoils, a portable permanent magnet, and custom-built NMR hardware. The measurement time is typically less than 30 min; the detection starts with cell separation, fixation, and pretreatment; then cells are incubated with monoclonal antibodies against the protein of interest coated with TCO-NHS (*trans*-cyclooctene), and then are reacted with synthesized Tz-MNP (magnetic nanoparticles); and finally, the sample is located in the coil to get detected [42]. The sensitivity and specificity of ovarian cancer and lung cancer diagnosis are more than 90% by means of a quadruple marker subset (MUC1 + EGFR + HER2 + EpCAM) (Figure 2) [43,44]. The quad marker detection of cancer cells in blood is much more sensitive than conventional EpCAM-based detection. The use of μNMR technology for direct measurements of rare CTCs in whole blood is quite creative, and the preclinical validation of a quadruple CTC marker signature harbors promising clinical utility. The key question is whether the CTC levels and characters would be beneficial to the clinical decision.

#### Aptamer-associated detection strategy

Aptamers are single-stranded DNA or RNA oligonucleotides that bind to target molecules with robust binding affinities. They are generated though repeated rounds of processes termed systematic evolution of ligands by exponential enrichment (SELEX) and bind to a wide range of target molecules, including extracellular ligands and cell surface proteins, small interfering RNAs (siRNAs), chemotherapeutic agents, cell toxins, and nanoparticles [45]. Moreover, man-made aptamers possess several advantages over natural-made antibodies. The man-made aptamers can be generated rapidly and conveniently according to various diagnostic and therapeutic needs and can be stored with long-term stability as dry powder or in solution and act with fast tissue penetration. These chemical properties make aptamers ideal candidates as probes for detection and targeted therapy and may replace the antibody in research, diagnostic methods, and therapeutics [46].

Huang et al. design multiple aptamers conjugated on Au-Ag nanorods, up to 80 fluorophore-labeled aptamers attached on a 12 nm × 56 nm nanorod [47]. This leads to an affinity at least 26-fold higher than the intrinsic affinity of the original aptamer probes, and the fluorescence signal increases by 300-fold compared with those labeled by the individual aptamer probe. Researchers have already designed a creative method to detect CTCs without a fluorochrome by taking advantage of aptamer technology [22]. Due to their plasmon resonance, when the gold nanoparticles come into proximity with one another, their absorption spectra and their scattering profiles change. Therefore, the color of aptamer-conjugated gold nanoparticles (ACGNPs) bound with targeted cells shifts while the unbound ACGNPs show no change, so the bare eyes as well as a colorimetric assay identify the color change and it is likely to be a very useful assay technique for point-of-care diagnostics.

Besides, aptamers have a wide application in tumor therapy. As aptamers are an excellent intermedium, they combine various organic molecules to form chimeras, for example, aptamer-antibody chimera, aptamer-protein chimera, aptamer-siRNA chimera, and aptamer-miRNA chimera and then combine with nanoparticle as a carrier to be absorbed by the CTCs to perform pharmacological action [46,48].

#### Magnetophoresis technology to separate CTCs

That the magnetic particle is drove to a certain direction in the magnetic field is defined as magnetophoresis, and the course can be compared with electrophoresis course, which is an efficient and high-throughput separation technology in recent years [49,50]. It is an easy, rapid, and accurate method to separate RNA and tumor cells [51-53]. Herein, we introduce a novel CTC separation device applying the lateral magnetophoresis principle designed by Kim et al. [23]. The device is mainly fabricated by two pieces of glass slides, and the core which consists of a ferromagnetic permalloy wire array is hidden between them. The device detects CTCs effectively and efficiently since experimental results indicate that the CTC microseparator isolates approximately 90% of CTCs spiked into blood samples with a flow rate of up to 5 ml/h and the purity of separated CTCs is 97%; the overall isolation procedure can be completed within 15 min for 200 μL of peripheral blood. Besides, clinical practice has demonstrated that it can monitor the therapeutic effect and recurrence of tumor.

The process is quite simple and only needs three steps: buffer injection only, sample and buffer at the same flow rate, and a second injection of buffer only. So the CTC microseparator platform is easy to establish and automate. The technology has many unique properties. Firstly, the separation method maintains intactness and contributed to further study about CTCs. Secondly, the simple procedure reduces CTC loss during the enrichment process, improving the sensitivity. Thirdly, the CTC microseparator can be simply applied for the separation of various CTCs by using other tumor-specific antibodies and changing the size of the magnetic nanobeads to optimize the magnetic force to detect specific tumor CTCs. As it can be combined with other advanced genetic detection methods (e.g., single-cell RT-PCR), it pushes the development of an automated platform for CTC-based cellular and molecular assays.

### The application of nanotechnology in CTC therapy

Nanoparticles have many unique and excellent physical properties, and we can take advantage of the properties to overcome the limitations of traditional diagnostic and therapeutic methods [54]. The nanoparticles applied in tumor therapy currently include liposomes, polymeric nanoparticles, protein nanoparticles, ceramic nanoparticles, metallic nanoparticles, and carbon nanotubes [55], but only a few are approved by the FDA, such as pegylated liposomal doxorubicin (Doxil in the USA and Caelyx outside the USA), liposomal daunorubicin (DaunoXome), non-pegylated liposomal doxorubicin (Myocet), and albumin-bound paclitaxel nanoparticles [56]. Faltas has already concluded some CTC therapeutic methods, but many are still in the experimental stage and there are many problems to be solved before being applied in routine clinical practice [57]. Herein, we introduce some novel methods for CTC therapy.

The nanoparticles and the halloysite nanotube fixed on the substrate significantly increase the number of combined selectin and tumor necrosis factor (TNF)-related apoptosis-inducing ligand (TRAIL), thus improving the capture efficiency for targeting CTCs [58,59]. Besides, TRAIL induces a death signal via the caspase pathway [60,61]. There is no significant effect of TRAIL on hematopoietic stem cells and other normal blood cells, but it induces apoptosis in a wide variety of cancer cells. Rana et al. design a capillary flow chamber by a bionic method [62]. The surface of the flow capillary chamber is functionalized with TRAIL and E-selectin and takes 1 h to kill 30% of the captured cells, but in the static condition, it will take 4 h to kill 30% of the cells. Different protein molecules could be applied in the device to capture various CTCs to induce apoptosis; the same technology can be applied in clinical practice to capture rare cells in the peripheral blood. The technology applied in clinical practice will significantly decrease the load of the disseminated tumor cells in cancer patients.

Besides, the microtube flow device proves to kill CTCs more efficiently if the specimen is pretreated with a particular drug. Clinical trials indicate that aspirin prevents the occurrence of colorectal cancer [63-66]. Aspirin treatment alone kills only about 3% of Colo205 cells when compared to untreated control. When cells pretreated with aspirin are perfused through the microtube flow device for 1 h and analyzed 18 h later, the kill rate is 44.32% while perfusing untreated cells for 1 h shows a kill rate of 17%. The kill rate of cells perfused through the microtube following aspirin pretreatment for 1 h is found to be similar to that of untreated cells perfused over the combined surface for 2 h. This means that the CTCs pretreated with aspirin can be killed more rapidly and the microtube functionalized with TRAIL and E-selectin can be combined with drugs to kill cancer cells to reduce the cells more quickly and efficiently [67].

Another novel nanodevice has been developed to kill CTCs. The strong absorbance of single-walled carbon nanotubes (SWCNTs) in 700 to 1,100-nm near-infrared (NIR) light can be used for optical stimulation of nanotubes inside living cells to afford various useful functions. When the nanotubes are coated with folacin, they can combine with tumor cells with folacin specially, and then consistent NIR light radiation causes cell death without harming receptor-free normal cells. So SWCNT combined with specific chemical matter under NIR light radiation is a novel way to convey drugs and treat tumors [68,69]. Neves et al. [70] design a SWCNT-annexin V (AV) conjugate by taking advantage of the phenomenon that AV combines specially with the anionic phospholipids expressed externally on the surface of tumor cells and endothelial cells that line the tumor vasculature. It kills most 4 T1 mouse mammary tumors for the majority of the animals by 11 days since the irradiation is at a wavelength of 980 nm. The combination of photothermal therapy with the immunoadjuvant cyclophosphamide results in an increased survival rate. Also, *in vivo* results suggest that the SWCNT-AV/NIR treatment is a promising approach to treat cancer. Moreover, Hossain et al. utilize iron oxide nanoparticles and bismuth nanoparticles to combine the folate receptor and then kill CTCs under X-ray radiation [71]. The systemic toxicity associated with conventional therapy may thus be significantly reduced in targeted photodynamic therapy, but a series of problems should be solved before entering clinical practice, such as the toxicity of nanoparticles and how to eliminate them in the body after use [72].

**Figure 3:**
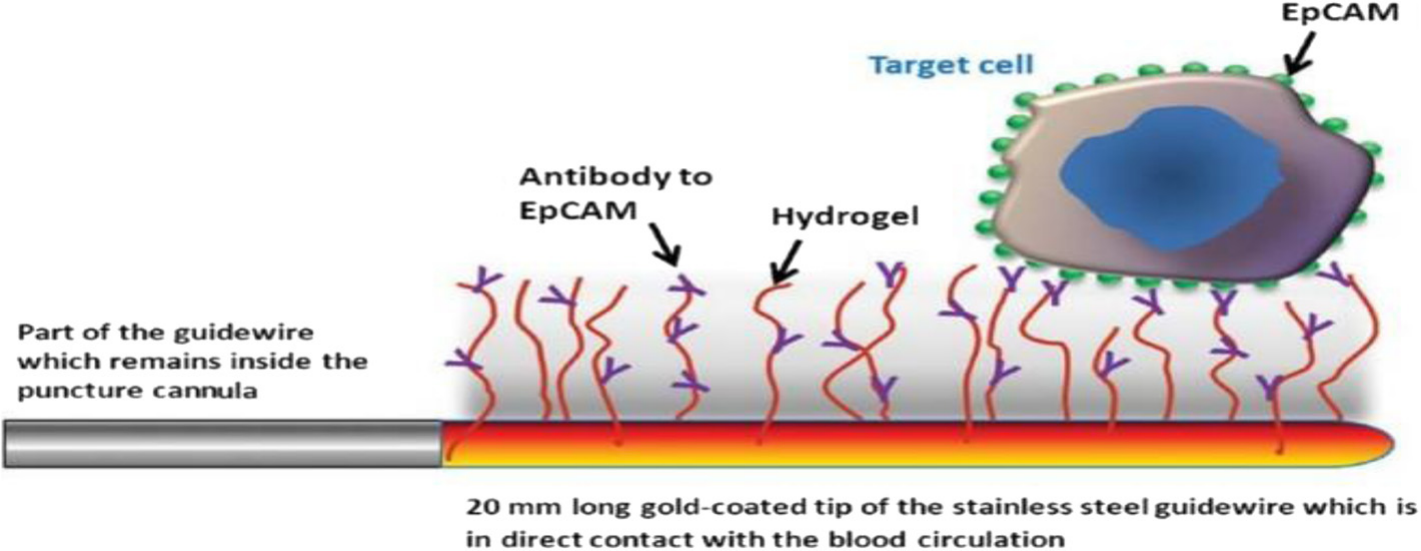
**Schematic drawing of the functionalized tip of the FSMW.** The gold-coated tip of the stainless steel captures CTCs in the circulating blood for 30 min. (Adapted from [73]).

One new trend of manipulating CTCs is to kill CTCs *in vivo*. Researchers have already developed a medical device, functionalized structured medical wire (FSMW), that offers opportunity of capturing CTCs from the circulating blood of cancer patients (Figure 3) [73]. The device is based on a stainless steel medical wire of 0.5 mm in diameter and 160 mm in length. The first 20 mm is plated with a thick gold layer, and a synthetic polycarboxylate and anti-EpCAM are attached to the gold layer. The EpCAM-functionalized FSMW surface dwells in the lumen of the vein for 30 min, and the total volume of blood that comes in contact with the FSMW is estimated to be 1.5 to 3 l. The capture rate of CTCs is 10/12 for patients with breast cancer with a median of 5.5 CTCs and 12/12 for patients with non-small lung cell cancer (NSCLC) with a median of 16 CTCs. As an *in vivo* device to capture CTCs, it is very unique and paves the way for future study. It is not only a way to capture CTCs but also offers a potential therapeutic trend which will initiate ‘dialysis therapy’ for cancer therapy just as dialysis therapy for renal failure.

As the CTCs shed from the primary tumor inconstantly, the blood samples extracted from the patients only reflected a temporal condition [6]. In order to realize consistent detection and treatment, the implantable vascular shunt device is an ideal design. Wojciechowski et al. have already used the device to capture CD34+ hematopoietic stem and progenitor cells (HSPCs) [74]. Similarly, we can design a vascular shunt to capture CTCs. The CTCs should be captured firstly and then induce apoptosis to decrease the CTC load and inhibit the tumor metastatic progress. Then we capture specific CTCs by means of different molecules, such as CD44, CK, and E-selectin. Apoptotic molecules including TRAIL [62,75], Fas-L [76-78], TNF [79,80], and liposomes with encapsulated siRNA [81] will make a difference in the kill of CTCs.

Scarberry et al. have demonstrated that targeted removal of migratory tumor cells by functionalized magnetic nanoparticles impedes metastasis and tumor progression [82]. They mix the magnetic nanoparticles (MNPs) functionalized with ephrin-A1 mimetic peptides selective for the EphA2 receptor with the peritoneal fluid withdrawn from female C57BL/6 mice injected with a murine ovarian cancer cell line. Then the processed peritoneal fluid is re-introduced into the peritoneal cavity. They found that tumor progression of the experimental group is 10.77 times slower than that of the control group which receives no intervention, and the median time to endpoint for the experimental group (49 days) is 32.4% longer than that for the control group (37 days), which indicates that the intervention is quite efficient. We envision that the technology could be applied to cancer patients. Firstly, the body liquid (including blood and peritoneal fluid) is pumped out and then mixes with the MNPs to discharge the malignant cells; the purged liquid is then returned to the body. Once the protocol is accomplished at a satisfying level, it shall be an amazing breakthrough in tumor therapy.

## Conclusions

At present, the most important applications of the CTC technology are the monitoring of the CTC account of cancer patients and evaluation of the metastasis of malignant tumor [6,7,83]. But the present devices are not ideal enough to meet the application needs because the problems of insufficient capture, low purity, and narrow detection spectrum still need to be addressed. Manipulating CTCs *in vivo* directly is an attractive direction; it denotes that the process can reduce tumor metastasis and even cut metastasis while dealing with the primary tumor, and it also means that we should overcome some great challenges: temporal heterogeneity of dissemination and sample size limitations for *in vivo* techniques [25], so much more effort should be paid to enhance the practical application of CTCs. Nanotechnology has already been studied a lot in CTCs, and it probably has a wider application in tumor diagnosis and treatment. We can design a long therapeutical guidewire by virtue of nanotechnology and CTC theory which is placed in the vessel by means of interventional methods. The surface of the guidewire is covered with various kinds of nanoparticles decorated with adhesion molecules and apoptosis-inducing ligands for the capture and kill of CTCs. In order to realize personal therapy, the nanoparticles can be modified according to the characteristics of the CTCs. The therapy outcomes can be quantified by comparing the number of CTCs before and after the process. If we keep the number of CTCs at quite a low level, patients can survive longer even if they live with tumor [7].

In a word, with the advent of translational medicine and multiple-discipline treatment (MDT), the basic research products about nanotechnology and CTCs are supposed to offer a better guide for clinical decision. Meanwhile, we should also pay attention on the side effects of nanotechnology while exploiting it. In the future, the use of nanotechnology for CTC detection and kill is promising, and it will offer a multifunctional, potable platform in the field of anticancer therapy.

## Competing interests

The authors declare that they have no competing interests.

## Authors' contributions

YG collected and reviewed the data and drafted the manuscript. ZY modified the draft in the first version and after revision. Both authors helped in drafting the manuscript. Both authors read and approved the final manuscript.
